# The History and Capabilities of Herbal Simples

**Published:** 1890-02-08

**Authors:** W. T. Fernie


### The History and Capabilities of Herbal Simples.

By W. T. Fernie, M.D.

(*Continued from page 226.*)

### IV.—THE APPLE.

No fruit has played such an eventful part in the world's history as the so-called apple. In the primitive Garden of Eden, as we have been taught from childhood to believe, were associated "Man's first disobedience and the fruit of that forbidden tree, whose mortal taste brought death into the world, and all our woe"; the golden apple adjudged by Paris to Venus, as the fairest goddess—*detur pulchiori*—led to the long Trojan War; Hercules had to procure the golden apple from the garden of the Hesperides, and to brave the anger of a sleepless dragon; it was by throwing an apple to the ruthless, but beautiful Atalanta that Hippomenes outstript her in the race for his life; Solomon desired to be comforted with apples; and the patriot William Tell shot with one arrow an apple from the head of his own bright boy, whilst reserving his second arrow for the heart of a tyrant. Later on, the apple did much to redeem its fair fame by proving to Sir Isaac Newton, the great universal law of gravitation. Nevertheless, we have to remember that the apple of former historic times was rather a typical, globular, handy fruit, of varying sort, than our Pyrus Malus, descended from the wild crab-tree, and belonging to the natural order of rosaceous plants. This sour wilding of our woods—so called to distinguish it from the cultivated sweeting,—furnishes verjuice, rich in tannin, and a most useful application to recent sprains. Says Christopher North, "to the senses of a schoolboy a green, sour crab is as a golden pippin, and more delicious than any pineapple; the tree which he climbs to pluck it seems to grow for him in the blissful Garden of Eden." The apples of the Jews were golden oranges; and now with ourselves, many fruits not the produce of the orchard are called apples, as the thorn apple, the pine apple, the love apple, the custard apple, etc. Dr. Prior tells us that the word apple took its origin from the Sanskrit *ap*—water, and *p'hal*—fruit; meaning water fruit. or juice-fruit, with which the Latin word *pomum*, an apple, from *poto*, to drink, exactly tallies. If this be so, our apple tree came originally from the East, long ages back.

Chemically, the apple is composed of vegetable fibre, albumen, sugar, gum, chlorophyll, malic acid, gallic acid, lime, and much water. Furthermore, the German analysts say that the apple contains a larger percentage of phosphorus than any other fruit or vegetable. This phosphorus is admirably adapted for renewing the essential nervous matter, lethicin, of the brain and spinal chord. It is perhaps for the same reason, rudely understood, that old Scandinavian traditions represent the apple as the food of the gods, who, when they felt themselves to be growing feeble and infirm, resorted to this fruit for renewing their powers of mind and body. Also, the acids of the apple are of signal use for men of sedentary habits, whose livers are sluggish in action; these acids serving to eliminate from the body noxious matters which, if retained, would make the brain heavy and dull, or bring about jaundice or skin eruptions, and other allied troubles. Some such an experience must have led to our custom of taking apple-sauce with roast pork, rich goose, and like dishes. The malic acid of ripe apples, either raw or cooked, will neutralise any excess of chalky matter engendered by eating too much meat. It is also the fact that such fresh fruits as the apple, the pear, and the plum, when taken ripe and without sugar, diminish acidity in the stomach rather than provoke it. Their vegetable salts and juices are converted into alkaline carbonates, which tend to counteract acidity. A good ripe, raw apple is one of the easiest of vegetable substances for the stomach to deal with, the whole process of its digestion being completed in eighty-five minutes. Gerard found that the "pulpe of roasted apples mixed in a wine-quart of faire water, and laboured together until it comes to be as apples and ale—which we call lambeswool —never faileth in certain diseases of the raines, which myself hath often proved, and gained thereby both crownes and credit." "The paring of an apple, cut somewhat thick, and the inside whereof is laid to hot, burning, or running eyes at night, when the party goes to bed; and is tied, or bound to the same, doth help the trouble very speedily, and contrary to expectation—an excellent secret." A poultice made of rotten apples is of very common use in Lincolnshire for the cure of weak or rheumatic eyes. Likewise in the Hotel des Invalides, at Paris, an apple poultice is used commonly for inflamed eyes, the apple being roasted, and its pulp applied over the eyes without any intervening substance. Long ago it was said apples do easily and speedily pass through the belly; therefore they do mollify the belly; and, for the same reason, a modern maxim teaches that—

To eat an apple going to bed,
The doctor then will beg his bread.

There was made, in Gerard's time, an ointment with the pulpe of apples and swine's grease and rose-water, which was used to beautifie the face and to take away the roughnesse of the skin, and which was called in the shops pomatum—from the apples, poma—whereof it was made. In England apples were sold up to the time of Henry VII. for as much as eighteenpence, and two shillings each. Apple-water is a valuable cooling drink during fever. To make this take two sound apples, peel them, remove the cores, and cut them into thin slices; add a small quantity of the yellow rind of lemon, just enough to give a flavour, and put these, with a little sugar if approved, into a jug; pour on a pint of boiling water, and when it is cold strain ready for use. In Silesia an apple is scraped from the top to cure diarrhœa, and from the bottom to cure costiveness. Caracioli, an Italian writer, declared that the only ripe fruit he met with in Britain was a *baked* apple. Coleridge said: "No man has lost all simplicity of character who retains a fondness for apple dumplings." So highly did good old Gerard esteem apples that he exhorted his readers to "forward, in the name of God, graft, set, and nourish up trees in every corner of your ground; the labour is small, the cost is nothing, the commodity is great: yourselves shall have plenty, the poor shall have somewhat in time of want to relieve their necessitie; and God shall reward your good mindes and diligence." The juice of the cultivated apple, made by fermentation into cider—which means, literally, strong drink—was pronounced by John Evelyn, in his "Pomona"—1729—to be, "in a word, the most wholesome drink in Europe, as specially sovereign against the scorbute, the stone, spleen, and what not." This beverage contains alcohol, gum, sugar, some mineral matters, and several acids, among which the malic predominates. There is on an average a little over 5 per cent. of alcohol in English cider. As a drink it is apt to become sour in the stomach of a gouty person, and then to provoke rheumatism, if taken habitually.

The term *malus*, as applied to the apple, is derived from the Greek *melo*, to cure. I remember, when a schoolboy, the following play on this word: *Malo*, I had rather be; *malo*, in an apple-tree; *malo*, than a wicked man; *malo*, in adversity. And again: *Mea mater mala est sus*, which, if literally translated, would mean—My mother is a wicked sow (what could be more unfilial?), but which, more correctly translated, signifies, *Mea*, run; *mater*, O, mother; *sus*, the sow; *est*, is eating; *mala*, our apples. In old Rome the public feasts began with eggs and ended with apples; hence arose the saying, "*ab ovo usque ad mala*," from first to last. As varieties of the apple mention is made in documents of the twelfth century of the pearmain and the costard, from the latter of which came the word costardmonger, as at first, a dealer in this fruit, and now corrupted to our costermonger, who trafficks in cheap, perishable articles of food on a handbarrow or with a donkey. Among many modern fanciful names given to different apples that of *varpneys* is known in some rural districts. Apples so called used to be sold four for a penny, and when a lad went to buy them, he would ask for a pennyworth of vour-a-pennies, which became shortened to vourpennies, and then to varpneys. As a spell against inconstancy apples have long been, and are still supposed by the credulous, to exercise an undoubted and remarkable potency. About the year 1562 a certain rector of St. Ives, in Cornwall, practised physic with milk and apples so successfully in many diseases, and so spread his reputation, that numerous sufferers came to him from all the neighbouring counties. Dr. Johnson knew a clergyman, of small income, who brought up a large family very reputably, the members of which he fed chiefly on apple dumplings. In England and in Germany the apple is thought to be potent against warts. Any such virtue, if it really exists, depends probably on the presence in the fruit of earthy salts. For, it is a fact, though not generally known, that magnesia, as occuring in ordinary Epsom salts, will certainly cure warts, and the disposition thereto. Just a few grains—from three to six—not enough to produce any sensible medicinal effect, taken once or twice a day, for three or four weeks, will surely dispel the warts.

